# Putting equity first: reorienting artificial intelligence for population-level postpartum depression prevention

**DOI:** 10.3389/fpubh.2026.1967813

**Published:** 2026-09-25

**Authors:** Ya-li Yan, Qian-qian Li, Meng Xu

**Affiliations:** 1 Department of Gynecology, Suqian Hospital of Nanjing Drum Tower Hospital Group, Suqian, China; 2 Department of Obstetrics, Suqian Hospital of Nanjing Drum Tower Hospital Group, Suqian, China

**Keywords:** artificial intelligence, digital public health, health equity, postpartum depression, public health prevention

## Abstract

Postpartum depression (PPD) affects 10–20% of new mothers globally, yet detection and treatment rates remain low, particularly in underserved populations. Artificial intelligence (AI)—here spanning supervised machine-learning prediction, natural-language and acoustic analysis, and generative conversational agents—is increasingly promoted as a solution, from risk prediction to chatbot-delivered therapy. Drawing on selected evidence from clinical informatics, digital mental health, public-health ethics, and implementation science, we argue that although AI may genuinely expand reach, its current trajectory, driven by technical feasibility rather than public-health value, could widen rather than narrow maternal mental-health inequities. This risk plays out across four domains: (1) algorithmic accuracy that does not translate into population outcomes; (2) training data that structurally exclude marginalized groups; (3) algorithmic care that may erode relational support; and (4) a regulatory vacuum that permits deployment without accountability. Distinguishing published evidence from our own recommendations, we conclude that AI for PPD prevention should be governed as a public good through equity-centered design, real-world evidence standards (including calibration and subgroup performance beyond AUC), tiered device oversight, layered data consent, and clearly specified safety pathways. We call for a global dialogue among policymakers, developers, health systems, researchers, and women with lived experience to operationalize these principles before large-scale deployment.

## 1 Introduction

Millions of new mothers experience postpartum depression (PPD) yearly, yet most are never screened, diagnosed, or treated. PPD denotes a major depressive episode with onset during pregnancy or the first postpartum year. Its distinctive features: symptoms are entangled with infant care, sleep disruption, and mother–infant bonding; stigma and childcare logistics suppress help-seeking; and severe cases can involve self-harm or thoughts of harming the infant, so PPD concerns the mother–infant dyad, not the mother alone (1–4). Global prevalence is 10–20%, higher in low- and middle-income countries (LMICs) and marginalized populations (5, 6). Terminology matters. Classically, universal prevention addresses whole populations, selective prevention high-risk groups, and indicated prevention people with early or subclinical signs; all aim to lower incidence. Screening is the systematic administration of instruments such as the Edinburgh Postnatal Depression Scale (EPDS) to identify possible cases; detection, referral, and treatment support follow as distinct steps. Most AI tools reviewed here operate at indicated prevention and at the screening–detection–referral interface, and none has yet been shown to lower PPD incidence at population scale. We retain “prevention” in the title for this aim while labeling each tool by its actual function; unless stated otherwise, claims below concern screening, detection, referral, and treatment support rather than demonstrated prevention of new cases. Control remains reactive for three reasons. Coverage is low: even in high-income systems fewer than half of primary-care facilities screen routinely, and instruments depend on women presenting and disclosing, missing those silenced by stigma, geography, or mistrust (7); the workforce is scarcest where need is greatest—in primary care and rural settings (8, 9).

Artificial intelligence (AI) here comprises three families: (i) supervised machine-learning (ML) risk-prediction models; (ii) natural-language processing (NLP), acoustic, and speech-emotion recognition applied to text or voice; and (iii) generative conversational agents, including chatbots and large language models (LLMs) delivering information or structured therapy; robotic and medical-imaging AI are excluded; they span the care continuum, from social-media risk detection to chatbot-delivered cognitive behavioral therapy (CBT) (10–18), and are widely framed as answers to workforce and coverage gaps. Yet one question remains underexplored for postpartum mental health, where outcomes affect mothers and infants, does AI produce measurable population-level benefit, or risks falling disproportionately on the women least able to bear them?

Two terms require definition. Equality means giving every woman the same input, such as one identical app; equity means allocating support by need so groups reach comparable outcomes, for instance pairing community health workers (CHWs) with an app in low-connectivity communities. By population-level we mean action covering a defined population, measuring results separately across its subgroups, and using those data to allocate resources; a single large study sample is not equivalent to equitable population coverage. We examine this question through selected evidence, organized around four tensions—the accuracy–outcome gap, the data-bias problem, the human-connection deficit, and the governance void—before proposing an equity-centered framework assigning responsibilities to policymakers, developers, health systems, and researchers.

Scope and selection of evidence. This is a critical Perspective, not a systematic review. We searched PubMed, Web of Science, and Scopus (2020–2026, retaining seminal earlier work), combining postpartum/perinatal depression with artificial intelligence, machine learning, chatbot, bias, equity, privacy, and governance; prioritized systematic reviews, randomized trials, and multicenter validation studies; and added ethics, regulatory, and implementation-science sources. We did not apply PRISMA screening or pool effect sizes. We distinguish published findings from recommendations, using “may,” “could,” and “current evidence suggests” where evidence is limited.

## 2 The accuracy–outcome gap: what AI predicts versus what populations need

PPD prediction models have produced strong laboratory results. Random-forest, gradient-boosting, and deep-learning models using electronic health records, perinatal and obstetric history, and medication data—sometimes combined with wearable sleep and activity signals—have reported area under the receiver-operating-characteristic curve (AUC) values of 0.75–0.90 (11, 19–25). Beyond structured records, social-media NLP has detected depression and suicidality in mental-health research, occasionally at a prodromal stage before clinical contact (12); perinatal NLP remains limited, extending to PPD symptom subtypes extracted from clinical notes (26) and automated sorting of online lay health content (27), while speech-emotion and facial-expression analysis has been piloted for affect assessment in telehealth. In principle, dynamic risk stratification could direct visits, education, and peer support toward higher-risk women rather than universal, resource-intensive approaches.

Strong discrimination, however, does not automatically translate into population benefit; three weaknesses recur. First, AUC alone is inadequate: it ranks cases against non-cases but conveys nothing about absolute risk. Sensitivity, specificity, predictive values, and calibration are also needed. With PPD prevalence of only 10–20%, even a sensitive, specific model can yield low PPV and many false positives at population scale, and a well-discriminating model can be badly miscalibrated after transport (28). Second, most studies remain retrospective and single-center, with little multicenter external validation and varying outcome definitions (29, 30), and few test outcomes that matter to populations—increased detection, shorter onset-to-treatment time, or reduced community incidence (11, 31). Third, screening carries an ethical trade-off: sensitivity and specificity determine who is labeled and routed to care, and an over-sensitive algorithm may pathologize normal mood variation.

We therefore recommend that evaluation progress from internal through multicenter external validation to community cluster-randomized or implementation studies whose endpoints include detection, treatment initiation, cost-effectiveness, and outcome distribution across subgroups to contemporary standards (28). Current evidence supports rigorous experimentation, not unrestrained deployment; performance is not health impact.

## 3 The data-bias problem: who gets counted, who gets missed

Public health’s core promise is reducing health inequities—systematic, avoidable differences between groups; AI in PPD prevention risks doing the opposite. Most prediction models and language tools are trained on predominantly white, English-speaking, higher-socioeconomic, digitally literate populations (32). A scoping review of ML for mental health among immigrants and racial/ethnic minorities found an early proof-of-concept stage: small studies, inconsistent validation, and little evaluation in populations at greatest risk (33). Tools built on high-income, majority-language data may miss the linguistic registers of low-income or non-English-speaking communities, and a review of perinatal AI in LMICs found almost no tools with equitable performance across subgroups (34).

Underrepresentation now has documented downstream consequences. In matched Black–White samples, language models predicted depression severity among White social-media users but largely failed among Black users, who express distress through different linguistic features (35). Suicide-death prediction models after mental-health visits showed 62% sensitivity for White versus 10% for Black patients, and weak performance for American Indian/Alaskan Native patients (36); five leading speech-recognition systems made roughly twice as many errors transcribing Black as White speakers, compromising voice-based screening (37). Development-data inequities thus translate into missed detection and risk assessment among groups with least alternative care.

The consequence is paradoxical: AI screening marketed as universal can produce a new digital divide, directing scarce resources toward groups already represented in training data while missing those with greatest unmet need. The divide is compounded by access—older, less-educated, rural, and migrant women may lack the devices, connectivity, or literacy to use AI services, and gendered technology barriers can further exclude the women most in need.

This bias is not a temporary glitch fixed by more data; it reflects structural inequities in who participates in research and whose outcomes are measured. We therefore recommend lifecycle action: proactive inclusion of underrepresented groups, pre-specified subgroup performance-gap limits, local adaptation where universal models fail, and post-deployment monitoring by stratum (Section 6, Table 1); we recommend treating equity as a design requirement, not an afterthought.

**Table 1:** Risk-tiered governance and operational requirements we recommend for AI in postpartum depression (PPD) prevention.

Tier (intended purpose; risk)	Representative tools	Required performance evidence (beyond AUC)	Bias monitoring and safety pathway
Tier I — Information and self-monitoring; low risk; generally not a medical device	Psychoeducation; mood/self-monitoring diary; generic signposting to care	Content accuracy, usability, and engagement; no individual diagnostic or risk claim	Accessibility across devices, bandwidth, and literacy; explicit notice it is not clinical care; always-visible route to human help
Tier II — Screening, risk prediction, and decision support; moderate-to-high risk; regulated as SaMD	ML risk scores; NLP/acoustic screening; clinician-facing CDSS	Sensitivity/specificity, subgroup-specific PPV/NPV, calibration, decision-curve/clinical-utility analysis, multicenter external validation (28)	Pre-specified acceptable subgroup performance gaps and scheduled bias audits; every high-risk result referred to a named clinician with a documented response target; human review of positives
Tier III — Autonomous therapy and crisis interaction; highest risk; most stringent SaMD oversight	Generative chatbots/LLMs delivering therapy; crisis dialogue	Comparative trials versus usual care; adherence, harms, and health-economic outcomes; calibration and subgroup performance	Independent real-time safety layer for self-harm, infant-harm, or psychosis; named trained responder, with minutes-level review for imminent risk and same-day contact for elevated risk; pre-planned fallback to crisis line, CHW, midwife, or peer supporter (40, 43); logged adverse events; rapid update or withdrawal

Required evidence and oversight increase with intended-purpose risk tier. SaMD, software as a medical device; PPV/NPV, positive/negative predictive value; CDSS, clinical decision-support system; CHW, community health worker.

## 4 The human-connection deficit: when care becomes algorithmic

For mild-to-moderate PPD, psychotherapy—particularly CBT and interpersonal therapy—is first-line, yet trained therapists are scarce, especially in underresourced settings (12, 34). AI conversational agents deliver structured CBT skills and mindfulness guidance by text or voice at any hour (14, 15). Clinical-benefit evidence is modest: a completed postpartum randomized trial found a small reduction in self-rated depressive (PHQ-9) symptoms but no between-group difference on the EPDS or anxiety (15); other completed perinatal evaluations support usability, acceptability, and screening reach rather than symptom reduction (38), and adapted WHO-guided chatbot programs remain under evaluation (39). they could form a first stepped-care tier for mild-to-moderate cases, reserving specialist care for complex presentations.

We caution against extrapolating to population-level replacement of human care. The perinatal chatbot evidence base remains weak: samples are small, durations short, adherence poor, and health-economic outcomes virtually absent (16, 40). PPD is not merely cognitive distortions amenable to standardized delivery; it is rooted in social isolation, birth trauma, unmet attachment needs, and identity disruption in early motherhood. The quasi-interpersonal rapport of a chatbot may offer temporary comfort yet cannot bear the weight of relational repair; we flag this as our interpretive judgment, not an established empirical finding.

The risk is that health systems deploy AI as a substitute for, rather than an adjunct to, human care: if chatbots displace midwives, CHWs, and peer supporters in the name of efficiency, they deepen rather than reduce isolation. A related concern is overmedicalization: ubiquitous screening may label normal postpartum mood variation, adaptive anxiety, and fatigue as high-risk, triggering unnecessary follow-up and reinforcing the pressure of the “perfect mother” (17, 41, 42). We recommend that AI handle repetitive screening and standardized information while preserving the human relationships at the core of perinatal care.

We therefore recommend a safety layer independent of the conversational model. On detecting suicidal ideation, self-harm, thoughts of harming the infant, or features of postpartum psychosis—a psychiatric emergency endangering mother and infant (43)—we recommend three simultaneous actions: a human-written message with localized crisis-line and emergency numbers; an alert to a designated trained responder (a named on-call clinician or supervised crisis team, never a generic inbox); and suspension of automated “therapy,” which must never supply methods of harm (40). We recommend pre-specified targets—synchronous review within minutes for imminent risk, same-day contact for elevated risk, at all hours—with documented backup when the first responder is unavailable. Both directions of error require planning: false positives need prompt review minimizing labeling and stigma; false negatives require that an AI “low-risk” output never exclude a woman from care, that multiple help routes stay open, and screening be repeated. Because raising sensitivity increases false positives, safety is architectural rather than model-level (40). When no professional can respond in time—common in underserved areas—we recommend a pre-planned fallback: repeated crisis-line and emergency offers; contact with a trained CHW, midwife, or peer supporter; and, only as a time-limited bridge, a constrained safety script (not open-ended therapy) that stays with the woman, re-escalates automatically, and can never close her case as resolved, conditional on a local plan to build human capacity.

## 5 The governance void: who is accountable when AI fails?

Beyond individual-level tools, AI is acquiring surveillance functions—anonymized, aggregate trend monitoring for resource allocation, not individual tracking. Integrating search queries, social-media discourse, help-seeking behavior, and geographic data can map distress patterns, locating high-burden areas and service gaps more finely than passive reporting.

This concentrates unprecedented data power over women’s inner lives. PPD data are exceptionally sensitive—emotional states, family conflict, suicidal ideation—and consent conditions are often opaque; agreeing to use a service is not agreeing to any secondary use. We recommend distinguishing at least five permissions: (i) receiving the AI service; (ii) research use; (iii) model training; (iv) commercial development; and (v) third-party sharing, each with separate, granular, revocable consent, data minimization, and plain-language explanation, not one click licensing all downstream uses (44). When users click “agree,” they rarely understand that data may feed training, commercial development, or external sharing; without public-interest governance, prevention tools could become covert surveillance over motherhood itself (32, 45).

The regulatory environment has not kept pace, and we recommend oversight proportionate to purpose and risk. Low-risk tools providing generic psychoeducation or self-monitoring may warrant only transparency and content-accuracy requirements; we recommend that tools which diagnose, triage, or autonomously deliver therapy—especially those interacting with users in crisis—be regulated as software as a medical device (SaMD), requiring pre-market registration, clinical validation, adverse-event monitoring, defined update protocols, and lifecycle accountability (46). Most current PPD tools have not undergone such evaluation: when an algorithm misses suicide risk, intimate data are breached, or a biased model withholds care, accountability falls into a vacuum. We recommend that clear governance frameworks precede, not follow, population deployment.

A legitimate counterargument is that demanding evidence and safeguards may slow innovation where no alternative exists. The question is how to innovate: technologies deployed without foundational safeguards can produce harm and inequity, whereas embedding fairness and accountability from the outset may make innovation trustworthy and durable.

These tensions converge on one question: how can AI’s promise be realized without exacerbating its risks? Our answer is an equity-centered implementation framework (Figure 1; Table 1) assigning responsibilities across four stakeholder groups.

**Figure 1:**
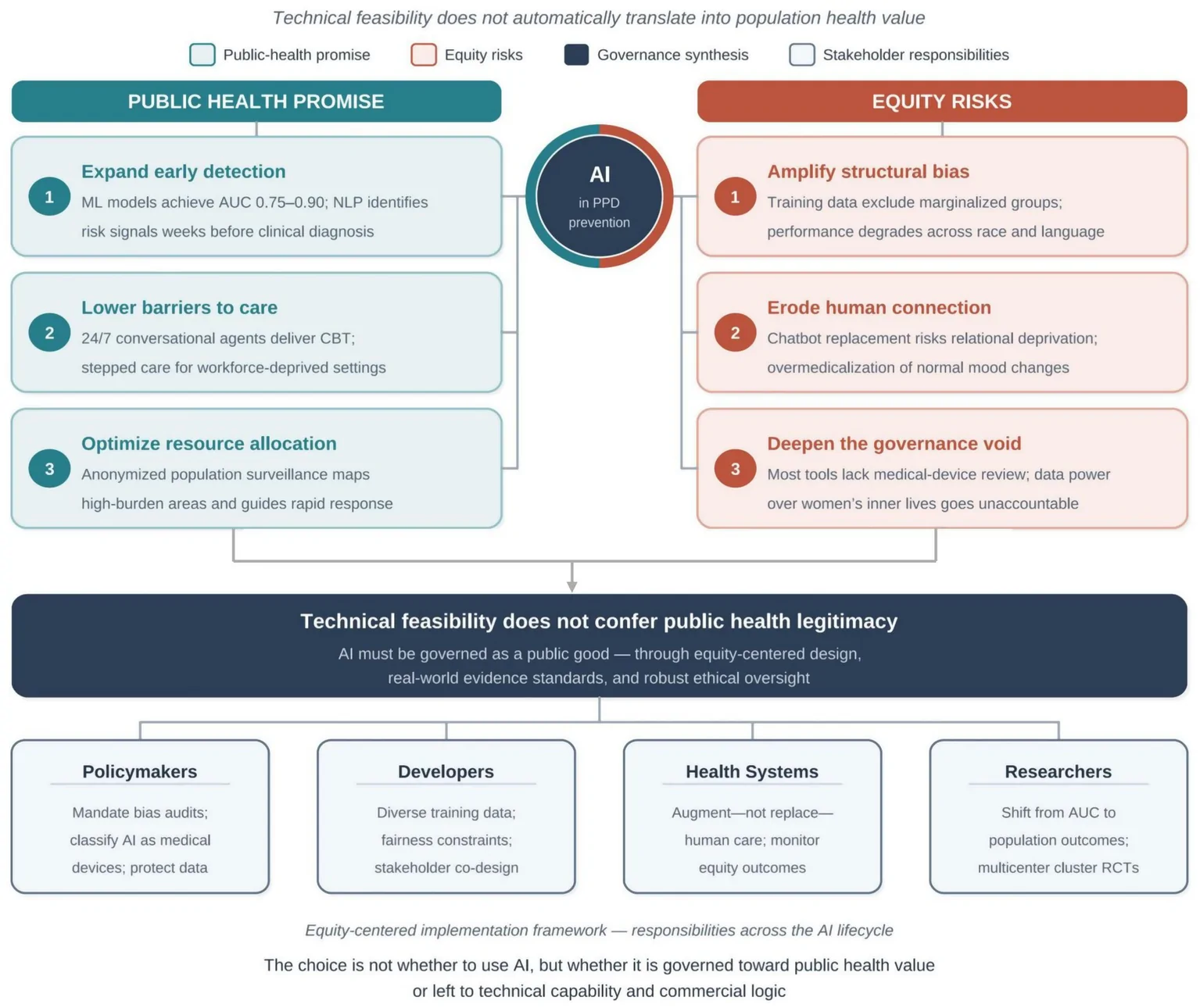
The dual potential of artificial intelligence in postpartum depression prevention. AI offers public-health promise—early detection, lower barriers to care, optimized resource allocation—but may amplify structural bias, erode human connection, and deepen the governance void. An equity-centered framework assigns responsibilities to policymakers, developers, health systems, and researchers so AI is governed as a public good, not by market-driven deployment.

## 6 A way forward: an equity-centered implementation framework

The choice is not whether to use AI, but whether it is governed toward public-health value or commercial logic. We propose four sets of responsibilities—our recommendations, not established standards—operationalized by risk tier in Table 1.

For policymakers, we recommend: routine bias audits for maternal-health AI tools, with publicly reported subgroup performance; adoption of the tiered device classification; and data-governance frameworks treating perinatal mental-health data as a public trust, with granular layered consent, data minimization, and restrictions on secondary commercial use.

For developers, we recommend: including diverse, underrepresented populations in training data from the outset; incorporating fairness constraints and documenting performance disparities by subgroup; building explainability into high-stakes models and interfaces that support rather than replace clinical judgment; and co-designing tools with clinicians, CHWs, and women with lived experience of PPD, who—consistent with patient-and-public-involvement principles—should take part across design, safety testing, and post-deployment monitoring rather than in a one-off consultation (47). These steps have proof of concept: in a widely used care-allocation algorithm, replacing a cost-based target with direct clinical-need measures cut its racial disparity in patient selection by over four-fifths (48).

For health systems, we recommend: positioning AI to augment human caregiving rather than cut costs; resourcing human-in-the-loop referral pathways for high-risk signals that meet the response targets above; investing in connectivity, devices, and digital literacy so AI does not exclude women without access; and monitoring equity outcomes after deployment, not only technical uptime.

For researchers, we recommend: shifting endpoints from technical metrics such as AUC toward population outcomes—detection, treatment initiation, cost-effectiveness, and equity in outcome distribution, including incidence reduction where feasible; prioritizing multicenter external validation and community cluster trials over retrospective single-center studies; reporting failures and null results as rigorously as successes; and reporting models completely, including calibration and subgroup performance (28).

Operation in low- and middle-income settings. The framework must function where connectivity, funding, and specialists are limited (34, 40). We recommend offline-first, low-bandwidth deployment via text, voice, or basic-phone channels; AI feeding task-sharing with CHWs, midwives, and peer supporters, who serve as the human interface routing referrals upward; local validation before scale-up rather than importing models unchanged; and staged rollout—pilot, validation, monitored expansion—paired with sustained financing and workforce development. Its most defensible role may be as a structured entry point strengthening available human care, not a standalone substitute. This human-supported, locally adapted model has empirical support: a culturally adapted WHO digital program guided by nonspecialist helpers yielded moderate, sustained reductions in depression and impairment among displaced Syrians in a randomized trial (49).

These responsibilities are interdependent: without mandates, developers will not prioritize equity; without representative data, systems cannot deploy fairly; without real-world evidence, regulators cannot govern intelligently. We recommend engaging researchers, clinicians, data scientists, ethicists, and—above all—women with lived experience from design onward.

Limitations of this Perspective. As a non-systematic narrative synthesis, evidence selection was interpretive and is itself concentrated in high-income, English-language settings, mirroring the gap we critique. Few studies report subgroup calibration or long-term population outcomes, and generative-agent evidence outpaces peer-reviewed validation, precluding pooling. Our proposals are expert judgments supported by adjacent evidence rather than approaches tested in perinatal AI; their feasibility and effects need prospective evaluation.

## 7 Conclusion

AI arrives at a critical juncture for PPD prevention. It could shift care from reactive, specialist-dependent management toward proactive, population-wide support through risk stratification, low-friction screening, treatment support, and aggregate surveillance—principally indicated prevention, whose effect on incidence remains unproven. This promise is not self-executing: tools that extend reach can entrench bias; efficiency that expands access can erode human connection; and data power that enables prevention can enable surveillance.

Technical feasibility does not confer public-health legitimacy. On current evidence, AI earns a place in PPD prevention only when equity, evidence, ethics, and humanistic care are embedded throughout research, development, and implementation. Inaction is itself a choice that defaults to market-driven deployment and leaves the women at greatest risk to bear the consequences. We recommend that policymakers steer AI toward genuine population-health value rather than treat technical novelty as its own justification.

## Data Availability

The original contributions presented in the study are included in the article/supplementary material, further inquiries can be directed to the corresponding author.

## References

[1] Richardson E Patterson R Meltzer-Brody S McClure R Tow A. Transformative therapies for depression: postpartum depression, major depressive disorder, and treatment-resistant depression. Annu Rev Med. (2025) 76:81–93. doi: 10.1146/annurev-med-050423-095712, 39527720

[2] Suryawanshi OT Pajai S. A comprehensive review on postpartum depression. Cureus. (2022) 14:e32745. doi: 10.7759/cureus.32745, 36686097 PMC9851410

[3] Tainaka H Takahashi N Nishimura T Okumura A Harada T Iwabuchi T . Long-term effect of persistent postpartum depression on children's psychological problems in childhood. J Affect Disord. (2022) 305:71–6. doi: 10.1016/j.jad.2022.02.061, 35219738 PMC11105964

[4] Suarez A Shraibman L Yakupova V. Long-term effects of maternal depression during postpartum and early parenthood period on child socioemotional development. Children. (2023) 10:1718. doi: 10.3390/children10101718, 37892381 PMC10605388

[5] Liu Y Zhang L Guo N Jiang H. Postpartum depression and postpartum post-traumatic stress disorder: prevalence and associated factors. BMC Psychiatry. (2021) 21:487. doi: 10.1186/s12888-021-03432-7, 34610797 PMC8491367

[6] Wells T. Postpartum depression: screening and collaborative management. Prim Care. (2023) 50:127–42. doi: 10.1016/j.pop.2022.10.011, 36822723

[7] Oliveira TA Luzetti G Rosalem MMA Mariani Neto C. Screening of perinatal depression using the Edinburgh postpartum depression scale. Rev Bras Ginecol Obstet. (2022) 44:452–7. doi: 10.1055/s-0042-1743095, 35253138 PMC9948039

[8] Putri AS Wurisastuti T Suryaputri IY Mubasyiroh R. Postpartum depression in young mothers in urban and rural Indonesia. J Prev Med Public Health. (2023) 56:272–81. doi: 10.3961/jpmph.22.534, 37287205 PMC10248106

[9] Sun M Cao F Peng J Tang J He Y Zeng Y . Prevalence and risk factors of postpartum depression among women in low-income developing rural areas: a cross-sectional study in China. Depress Anxiety. (2024) 2024:8841423. doi: 10.1155/2024/8841423, 40226729 PMC11918501

[10] Xia J Chen C Lu X Zhang T Wang T Wang Q . Artificial intelligence-oriented predictive model for the risk of postpartum depression: a systematic review. Front Public Health. (2025) 13:1631705. doi: 10.3389/fpubh.2025.1631705, 40969643 PMC12440769

[11] Qi W Wang Y Wang Y Huang S Li C Jin H . Prediction of postpartum depression in women: development and validation of multiple machine learning models. J Transl Med. (2025) 23:291. doi: 10.1186/s12967-025-06289-6, 40055720 PMC11887113

[12] Lee EE Torous J De Choudhury M Depp CA Graham SA Kim HC Artificial intelligence for mental health care: clinical applications, barriers, facilitators, and artificial wisdom. Biol Psychiatry Cogn Neurosci Neuroimaging (2021) 6:856–864. doi:doi: 10.1016/j.bpsc.2021.02.001 PMC8349367 33571718

[13] Dehbozorgi R Zangeneh S Khooshab E Nia DH Hanif HR Samian P . The application of artificial intelligence in the field of mental health: a systematic review. BMC Psychiatry. (2025) 25:132. doi: 10.1186/s12888-025-06483-2, 39953464 PMC11829440

[14] Alam S Sohail SS. Integrating ChatGPT: enhancing postpartum mental healthcare with artificial intelligence (AI) support. Digit Health. (2024) 10:20552076241295565. doi: 10.1177/20552076241295565, 39655059 PMC11626650

[15] Suharwardy S Ramachandran M Leonard SA Gunaseelan A Lyell DJ Darcy A . Feasibility and impact of a mental health chatbot on postpartum mental health: a randomized controlled trial. AJOG Glob Rep. (2023) 3:100165. doi: 10.1016/j.xagr.2023.100165, 37560011 PMC10407813

[16] Boucher EM Harake NR Ward HE Stoeckl SE Vargas J Minkel J . Artificially intelligent chatbots in digital mental health interventions: a review. Expert Rev Med Devices. (2021) 18:37–49. doi: 10.1080/17434440.2021.2013200, 34872429

[17] Wang X Sun M Cui J Liu Y Liu F Li J. Risk-prediction models for postpartum depression: assessment of risk of bias using PROBAST+AI. Midwifery. (2026) 159:104778. doi: 10.1016/j.midw.2026.104778, 42048745

[18] Wang Y Yan P Wang G Liu Y Xiang J Song Y . Trajectory on postpartum depression of Chinese women and the risk prediction models: a machine-learning based three-wave follow-up research. J Affect Disord. (2024) 365:185–92. doi: 10.1016/j.jad.2024.08.074, 39154983

[19] Matsuo S Ushida T Emoto R Moriyama Y Iitani Y Nakamura N . Machine learning prediction models for postpartum depression: a multicenter study in Japan. J Obstet Gynaecol Res. (2022) 48:1775–85. doi: 10.1111/jog.15266, 35438215

[20] Cellini P Pigoni A Delvecchio G Moltrasio C Brambilla P. Machine learning in the prediction of postpartum depression: a review. J Affect Disord. (2022) 309:350–7. doi: 10.1016/j.jad.2022.04.093, 35460742

[21] Zhong M Zhang H Yu C Jiang J Duan X. Application of machine learning in predicting the risk of postpartum depression: a systematic review. J Affect Disord. (2022) 318:364–79. doi: 10.1016/j.jad.2022.08.070, 36055532

[22] Huang X Zhang L Zhang C Li J Li C. Postpartum depression risk prediction using explainable machine learning algorithms. Front Med. (2025) 12:1565374. doi: 10.3389/fmed.2025.1565374, 40852354 PMC12367785

[23] Qasrawi R Amro M VicunaPolo S Abu Al-Halawa D Agha H Abu Seir R . Machine learning techniques for predicting depression and anxiety in pregnant and postpartum women during the COVID-19 pandemic: a cross-sectional regional study. F1000Res. (2022) 11:390. doi: 10.12688/f1000research.110090.1, 36111217 PMC9445566

[24] Zhang X Bao J Ye J Qiu X Pan Y. Machine learning-based methods for predicting postpartum depression: a review. Artif Intell Med. (2026) 176:103410. doi: 10.1016/j.artmed.2026.103410, 41861439

[25] Zhang Y Wang S Hermann A Joly R Pathak J. Development and validation of a machine learning algorithm for predicting the risk of postpartum depression among pregnant women. J Affect Disord. (2021) 279:1–8. doi: 10.1016/j.jad.2020.09.113, 33035748 PMC7738412

[26] Adekkanattu P Vekaria V Zhang Y Patra BG Liang P Sharko M . Identifying postpartum depression subtypes using natural language processing and clinical notes. BMJ Ment Health. (2026) 29:e302066. doi: 10.1136/bmjment-2025-302066, 41871883 PMC13034234

[27] Patra BG Sun Z Cheng Z Kumar P Altammami A Liu Y . Automated classification of lay health articles using natural language processing: a case study on pregnancy health and postpartum depression. Front Psych. (2023) 14:1258887. doi: 10.3389/fpsyt.2023.1258887, 38053538 PMC10694448

[28] Collins GS Moons KGM Dhiman P Riley RD Beam AL Van Calster B TRIPOD+AI statement: updated guidance for reporting clinical prediction models that use regression or machine learning methods. BMJ (2024) 385:e078378. doi:doi: 10.1136/bmj-2023-078378 PMC11019967 38626948

[29] Chiang MA Coll L Pollo-Cattaneo MF Chatterjee P. A systematic review on artificial intelligence-based clinical decision support systems in depression. Annu Int Conf IEEE Eng Med Biol Soc. (2025) 2025:1–7. doi: 10.1109/EMBC58623.2025.11253470, 41337284

[30] Ruger-Navarrete A Gomez-Ferrera M Merida-Yanez B Vazquez-Lara JM Gomez-Salgado J Garcia-Oliva S . Artificial intelligence in the prevention and early detection of postpartum depression: a systematic review and meta-analysis. Front Psych. (2025) 16:1734102. doi: 10.3389/fpsyt.2025.1734102, 41641079 PMC12864048

[31] Reading Turchioe M Desai P Reza Z Benda N. Toward an ethical framework for patient-facing artificial intelligence in clinical settings. Stud Health Technol Inform. (2026) 336:655–9. doi: 10.3233/SHTI260252, 42174927

[32] El Arab RA Al Moosa OA Albahrani Z Alkhalil I Somerville J Abuadas F. Integrating artificial intelligence into perinatal care pathways: a scoping review of reviews of applications, outcomes, and equity. Nurs Rep. (2025) 15:281. doi: 10.3390/nursrep15080281, 40863668 PMC12388636

[33] Park KK Saleem M Al-Garadi MA Ahmed A. Machine learning applications in studying mental health among immigrants and racial and ethnic minorities: an exploratory scoping review. BMC Med Inform Decis Mak. (2024) 24:298. doi: 10.1186/s12911-024-02663-4, 39390562 PMC11468366

[34] Anaduaka US Oladosu AO Katsande S Frempong CS Awuku-Amador S. Leveraging artificial intelligence in the prediction, diagnosis and treatment of depression and anxiety among perinatal women in low- and middle-income countries: a systematic review. BMJ Ment Health. (2025) 28:e301445. doi: 10.1136/bmjment-2024-301445, 40234194 PMC12001354

[35] Rai S Stade EC Giorgi S Francisco A Ungar LH Curtis B . Key language markers of depression on social media depend on race. Proc Natl Acad Sci USA. (2024) 121:e2319837121. doi: 10.1073/pnas.2319837121, 38530887 PMC10998627

[36] Coley RY Johnson E Simon GE Cruz M Shortreed SM. Racial/ethnic disparities in the performance of prediction models for death by suicide after mental health visits. JAMA Psychiatry. (2021) 78:726–34. doi: 10.1001/jamapsychiatry.2021.0493, 33909019 PMC8082428

[37] Koenecke A Nam A Lake E Nudell J Quartey M Mengesha Z . Racial disparities in automated speech recognition. Proc Natl Acad Sci USA. (2020) 117:7684–9. doi: 10.1073/pnas.1915768117, 32205437 PMC7149386

[38] Peters CJ Aldana Lainez V Clark K Jasczynski M Nguyen QC Norell EM. Using an AI-powered Mobile application Chatbot to address maternal depression indicators and inquiries in the perinatal and postpartum periods: a multimethod analysis. Inquiry. (2026) 63:469580261417580. doi: 10.1177/00469580261417580, 41607314 PMC12855737

[39] Rizzi S Fietta V Gios L Poggianella S Pavesi MC De Luca C . A chatbot-based version of a World Health Organization-validated intervention (self-help plus) for stress management in pregnant women: protocol for a usability study. JMIR Res Protoc. (2025) 14:e53891. doi: 10.2196/53891, 40590604 PMC12357124

[40] Luxton DD. Ethical implications of conversational agents in global public health. Bull World Health Organ. (2020) 98:285–7. doi: 10.2471/BLT.19.237636, 32284654 PMC7133471

[41] Alkhateeb M Nayeem A Ahmed A Alsahli M Sheikh J Abd-Alrazaq A. AI for detecting and predicting postpartum depression: scoping review. J Med Internet Res. (2026) 28:e77376. doi: 10.2196/77376, 41505715 PMC12782538

[42] Sezgin E Chekeni F Lee J Keim S. Clinical accuracy of large language models and Google search responses to postpartum depression questions: cross-sectional study. J Med Internet Res. (2023) 25:e49240. doi: 10.2196/49240, 37695668 PMC10520763

[43] Friedman SH Reed E Ross NE. Postpartum Psychosis. Curr Psychiatry Rep. (2023) 25:65–72. doi: 10.1007/s11920-022-01406-4, 36637712 PMC9838449

[44] Jacobson NC Bentley KH Walton A Wang SB Fortgang RG Millner AJ . Ethical dilemmas posed by mobile health and machine learning in psychiatry research. Bull World Health Organ. (2020) 98:270–6. doi: 10.2471/BLT.19.237107, 32284651 PMC7133483

[45] Yang H Lin DM. Conversational AI for perinatal mental health: promise, limits, and a human-AI stepped-care framework. Front Psych. (2026) 17:1847854. doi: 10.3389/fpsyt.2026.1847854, 42211192 PMC13212461

[46] Liu Y Yu W Dillon T. Regulatory responses and approval status of artificial intelligence medical devices with a focus on China. NPJ Digit Med. (2024) 7:255. doi: 10.1038/s41746-024-01254-x, 39294318 PMC11410966

[47] Zimmermann M Yonkers KA Tabb K Schaefer A Peacock-Chambers E Clare CA . Developing personas to inform the design of digital interventions for perinatal mental health. JAMIA Open. (2024) 7:ooae112. doi: 10.1093/jamiaopen/ooae112, 39494447 PMC11528508

[48] Obermeyer Z Powers B Vogeli C Mullainathan S. Dissecting racial bias in an algorithm used to manage the health of populations. Science. (2019) 366:447–53. doi: 10.1126/science.aax2342, 31649194

[49] Cuijpers P Heim E Abi Ramia J Burchert S Carswell K Cornelisz I . Effects of a WHO-guided digital health intervention for depression in Syrian refugees in Lebanon: a randomized controlled trial. PLoS Med. (2022) 19:e1004025. doi: 10.1371/journal.pmed.1004025, 35737665 PMC9223343

